# Screening of diverse Psylloidea species in Taiwan reveals the presence of both known and potentially novel “*Candidatus* Liberibacter” species in multiple psyllid lineages

**DOI:** 10.1128/spectrum.01228-25

**Published:** 2025-06-30

**Authors:** Reun-Ping Goh, Yi-Chang Liao, Man-Miao Yang, Chia-Ching Chu

**Affiliations:** 1 Department of Plant Pathology, National Chung Hsing University 34916, Taichung, Taiwan; 2 Department of Entomology, University California Riverside 385623 , Riverside, California, USA; 3 Department of Entomology, National Chung Hsing University 593978, Taichung, Taiwan; USDA-ARS San Joaquin Valley Agricultural Sciences Center, Parlier, California, USA

**Keywords:** insect-microbe association, phylogenetic analysis, plant disease, fastidious bacteria

## Abstract

Psyllids (Psylloidea) are host-specific, phloem-feeding insects that are associated with the transmission of destructive plant diseases caused by the bacterial genus “*Candidatus* Liberibacter”. While psyllid-liberibacter interactions have been studied in great detail, most research mainly focused on species associated with important crop diseases. Therefore, a more general understanding of the associations between these two groups of organisms remains limited. The present study investigated the occurrence of infection by “*Ca*. Liberibacter” across undercharacterized psyllid lineages in Taiwan. Samples of 46 psyllid species, representing six of the seven known Psylloidea families from host plants spanning 22 families, were obtained. The presence of “*Ca*. Liberibacter” in adult psyllid DNA samples was determined by PCRs targeting 16S rDNA of “*Ca*. Liberibacter”. PCR tests amplifying mitochondrial 16S DNA of the psyllids were also carried out for quality confirmation. The assays detected “*Ca*. Liberibacter” in five of the psyllid species tested, namely *Calophya nigridorsalis*, *Homotoma radiata*, *Cacopsylla tobirae*, *Epipsylla albolineata*, and *Trioza quadrimaculata*. BLASTn searches and phylogenetic analysis on near-full-length 16S rDNA sequences identified the strain detected in *C. tobirae* as “*Ca*. Liberibacter europaeus” and showed that *E. albolineata* carried a strain closely related to a “*Ca*. Liberibacter” strain found in Bhutan, while the remaining detected strains could potentially be novel “*Ca*. Liberibacter” species. The findings from this study showed that a large diversity of “*Ca*. Liberibacter” could exist among underexplored psyllid and plant species, opening new avenues for investigating the ecology of “*Ca*. Liberibacter”.

**IMPORTANCE:**

Bacteria of the genus “*Candidatus* Liberibacter” can cause some of the most devastating plant diseases. Gaining a broader perspective on the diversity of associations between these bacteria and their psyllid vectors is crucial for both fundamental and applicative purposes. By taking advantage of the biological diversity in Taiwan, the present study conducted one of the broadest surveys on the presence of “*Ca*. Liberibacter” in psyllids, in terms of the diversities of the psyllids examined. The data from this work indicated that previously unknown “*Ca*. Liberibacter” genotypes and perhaps even novel species may be more prevalent among psyllid species than previously known. These findings highlight the potential importance of exploring psyllid-liberibacter associations from a broader ecological perspective.

## OBSERVATION

“*Candidatus* Liberibacter” is a genus of Gram-negative bacteria associated with plants (1, 2). Most members of this genus remain unavailable in pure culture, and several species are associated with devastating plant diseases affecting crop production (2–5). Pathogenic “*Ca*. Liberibacter” species like “*Ca*. Liberibacter asiaticus” and “*Ca*. Liberibacter solanacearum” can invade the phloem of the host plants and cause destructive diseases such as citrus greening and potato zebra chip (3, 4). There are also “*Ca*. Liberibacter” species or strains that behave as non-pathogenic endophytes (6, 7).

An interesting feature of “*Ca*. Liberibacter” is that almost all its species are reportedly transmitted by phloem-feeding psyllids (superfamily Psylloidea) (2), which are currently classified into families Aphalaridae, Calophyidae, Carsidaridae, Liviidae, Mastigimatidae, Psyllidae, and Triozidae (8). Psyllids transmit “*Ca*. Liberibacter” in a circulative, persistent, and propagative manner (9). Although extensive studies have been conducted on “*Ca*. Liberibacter” transmission processes and their repercussions on vector psyllids (2, 9–11), understanding of psyllid-liberibacter associations from a broader ecological perspective remains limited, mainly due to the fact that most relevant studies to date have focused on psyllid species associated with major crops of limited plant families (2, 9–11). Currently, around 4,000 psyllid species are known across more than 200 genera worldwide (8, 12). Given that all major known vectors of “*Ca*. Liberibacter” belong to Psylloidea (2), it is plausible that many unidentified psyllid-liberibacter exist among other species. Taiwan is known to host many psyllid species feeding on plants of distinct lineages (13–15), and recent studies on the island have revealed “*Ca*. Liberibacter” strains other than well-known pathogenic species (16, 17). By taking advantage of Taiwan’s rich biodiversity, we investigated the occurrence of infection by recognized or unknown species of “*Ca*. Liberibacter” in underexplored members of Psylloidea. Samples of 46 psyllid species belonging to six recognized families were obtained. Adult psyllids were collected during 2017–2024 (Table 1), and the host plants of these psyllids encompassed 22 plant families (Table 1). Among them, only one unpublished *Trioza* species (*Trioza* sp. A) was collected from two plant species (*Helicia cochinchinensis* and *H*. *obovatifolia*; Table 1); all other psyllid species were each collected from a single plant species. After collection, each psyllid’s gender was determined, and every insect was placed separately in a microcentrifuge tube and stored at −80°C. Psyllid DNA samples were extracted from single psyllids using the DNeasy Blood and Tissue Kit (Qiagen; supplemental methods). A total of 406 DNA samples were extracted for testing.

**TABLE 1:** Information of the psyllid species tested in this study

Psyllid family	Scientific name	Family of host plant	Host plant species	Year of sampling	Locality	No. of samples tested (M + F^*a*^)
Aphalaridae	*Blastopsylla occidentalis*	Myrtaceae	*Eucalyptus robusta*	2018	Taipei	3 + 5
	*Cornegenapsylla sinica*	Sapindaceae	*Dimocarpus longan*	2018	Taichung	6 + 5
	*Ctenarytaina insularis*	Myrtaceae	*Syzygium samarangense*	2020	Chiayi	10 + 10
	*Togepsylla takahashii*	Lauraceae	*Litsea hypophaea*	2017	Nantou	3 + 5
Calophyidae	*Calophya mangiferae*	Anacardiaceae	*Mangifera indica*	2018	Taichung	6 + 4
	*Calophya nigridorsalis*	Anacardiaceae	*Toxicodendron succedaneum*	2018	Taichung	6 + 10
Carsidaridae	*Dynopsylla pinnativena*	Moraceae	*Ficus nervosa*	2022	Chiayi	5 + 5
	*Homotoma radiata*	Moraceae	*Ficus superba*	2018	Nantou	2 + 8
	*Macrohomotoma robusta*	Moraceae	*Ficus vasculosa*	2018	Pingtung	3 + 5
	*Mesohomotoma camphorae*	Malvaceae	*Hibiscus mutabilis*	2018	Miaoli	4 + 4
Liviidae	*Paurocephala chonchaiensis*	Moraceae	*Ficus erecta*	2017	Miaoli	3 + 6
	*Paurocephala sauteri*	Moraceae	*Morus australis*	2017	Nantou	5 + 4
	*Paurocephala trematos*	Cannabaceae	*Trema orientale*	2017	Nantou	3 + 3
Psyllidae	*Anomoneura taiwanica*	Moraceae	*Morus australis*	2018	Taoyuan	4 + 5
	*Cacopsylla chinensis*	Rosaceae	*Pyrus pyrifolia*	2022	Nantou	5 + 5
	*Cacopsylla coccinea*	Lardizabalaceae	*Stauntonia obovatifoliola*	2018	Hsinchu	5 + 4
	*Cacopsylla schefflerae*	Araliaceae	*Schefflera octophylla*	2017	Taipei	4 + 5
	*Cacopsylla tobirae*	Pittosporaceae	*Pittosporum tobira*	2017	Hsinchu	2 + 3
	*Epipsylla albolineata*	Fabaceae	*Mucuna macrocarpa*	2017	Taichung	5 + 6
	*Epipsylla* sp*.*	Fabaceae	*Derris taiwaniana*	2022	Chiayi	5 + 5
	*Euphalerus tzuensis*	Lauraceae	*Actinodaphne acuminata*	2018	Taichung	3 + 3
	*Heteropsylla cubana*	Fabaceae	*Leucaena leucocephala*	2018	Taichung	4 + 4
	*Psylla deflua*	Lauraceae	*Neolitsea acuminatissima*	2018	Taichung	4 + 4
	*Psylla eriobotryae*	Rosaceae	*Eriobotrya deflexa*	2018	Taichung	4 + 5
	*Psylla tetrapanaxae*	Araliaceae	*Tetrapanax papyrifer*	2017	Nantou	5 + 4
Triozidae	*Baeoalitriozus yangi*	Ebenaceae	*Diospyros ferrea*	2017	Taichung	6 + 6
	*Cecidotrioza epica*	Symplocaceae	*Symplocos lancifolia*	2017	Taichung	3 + 3
	*Leptotrioza tutcheriae*	Theaceae	*Pyrenaria microcarpa*	2017	Hsinchu	4 + 1
				2020	Nantou	1 + 4
	*Leptynoptera sulfurea*	Calophyllaceae	*Calophyllum inophyllum*	2017	Taichung	2 + 3
	*Neotrioza shuiliensis*	Lauraceae	*Machilus japonicus*	2018	Taichung	1 + 8
	*Pauropsylla triozoptera*	Moraceae	*Ficus ampelos*	2018	Taichung	5 + 4
	*Petalolyma vittata*	Aquifoliaceae	*Ilex ficoidea*	2017	Nantou	3 + 1
	*Siphonaleyrodes formosanus*	Lauraceae	*Cinnamomum reticulatum*	2018	Pingtung	2 + 2
	*Trioza acuminatissima*	Lauraceae	*Neolitsea acuminatissima*	2018	Taichung	3 + 5
	*Trioza beilschmiediae*	Lauraceae	*Beilschmiedia erythrophloia*	2018	Taoyuan	5 + 5
	*Trioza caseariae*	Salicaceae	*Casearia membranacea*	2017	Nantou	4 + 4
	*Trioza exoterica*	Lauraceae	*Cryptocarya chinensis*	2020	Nantou	3 + 2
	*Trioza neolitseacola*	Lauraceae	*Neolitsea variabillima*	2018	Taoyuan	4 + 4
	*Trioza neolitseae*	Euphorbiaceae	*Mallotus philippensis*	2017	Taipei	5 + 5
	*Trioza outeiensis*	Myrtaceae	*Decaspermum gracilentum*	2018	Pingtung	4 + 4
	*Trioza quadrimaculata*	Fagaceae	*Castanopsis cuspidata*	2024	Taoyuan	3 + 5
	*Trioza resupina*	Myrtaceae	*Syzygium jambos*	2017	Taichung	4 + 5
	*Trioza sozanica*	Daphniphyllaceae	*Daphniphyllum pentandrum*	2017	Nantou	2 + 4
	*Trioza* sp*. A*	Proteaceae	*Helicia cochinchinensis*	2018	Hsinchu	4 + 2
			*Helicia obovatifolia*	2018	Taichung	2 + 0
	*Trioza* sp. B	Lauraceae	*Cinnamomum osmophloeum*	2020	Chiayi	4 + 9
	*Trioza* sp*.* C	Lauraceae	*Machilus thunbergii*	2024	Taoyuan	5 + 5

^*a*^ M: male; F: female.

Prior to “*Ca*. Liberibacter” detection, PCRs using primers Chiar16SF and Chiar16SR (18) (Table S1) targeting Hexapoda mitochondrial 16S rDNA were conducted using the GoTaq Green Master Mix (Promega) to confirm the quality of the DNA samples (supplemental methods), and all samples amplified the expected products. For “*Ca*. Liberibacter” detection, 1 µL of undiluted DNA samples (on average 83.5 ng per reaction) was subjected to PCR using primer pair LG774F/LG1463Rm targeting the 16S rDNA of recognized “*Ca*. Liberibacter” species (genus specific; Table S1). Among them, LG774 was designed in a previous study (6), while LG1463Rm was modified from LG1463R (Table S1; supplemental methods), a reverse primer designed in the same study as LG774. DNA samples of “*Ca*. Liberibacter asiaticus”-infected Ponkan (*Citrus reticulata* Blanco cv. Ponkan) leaves were used as positive controls. PCR assays were conducted using the GoTaq Green Master Mix, and samples producing amplicons with the expected size (~690 bp) were subsequently amplified using primers 27F (19) (Table S1) and LG1463Rm to obtain near-full-length 16S rDNA sequences while excluding samples that produced non-16S rDNA amplicons with sizes near 690 bp. For samples that produced faint bands with the expected size after amplification with 27F and LG1463Rm, the PCR products were cloned using the pGEM-T Easy Vector System (Promega) and subjected to screening (supplemental material). Eventually, only psyllid species with samples that produced prominent and specific bands in both initial PCR tests using LG774/LG1463Rm and 27F/LG1463Rm were found to contain “*Ca*. Liberibacter”; in all these species, at least one sample’s PCR product (one to five) underwent successful sequencing (Table S2), and only one type of sequence was detected in each species. To ensure that the peak signals represented true signals and not artifacts from non-specific amplification, one PCR product from each species was also cloned into pGEM-T Easy and sequenced.

**Fig 1:**
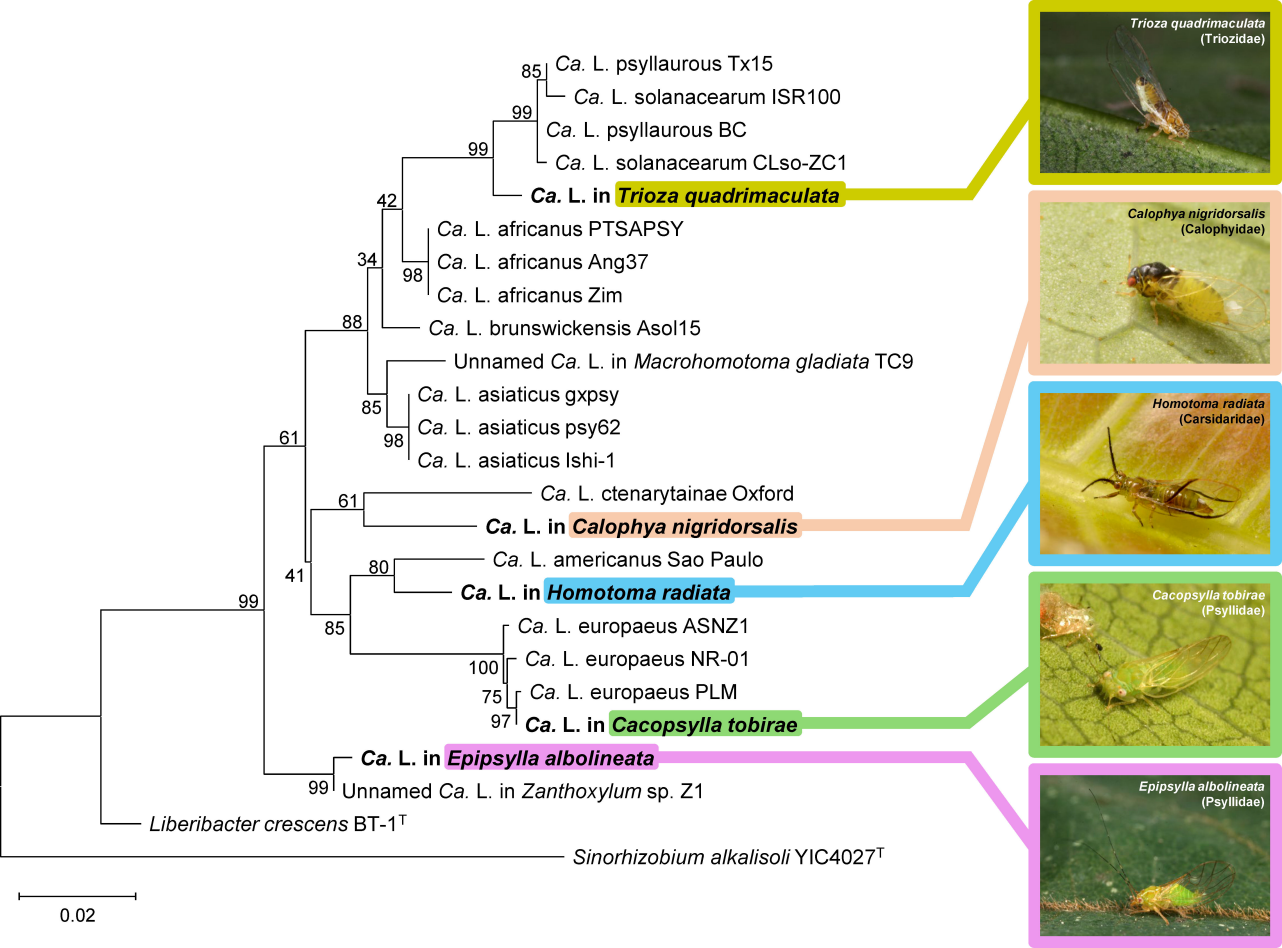
Maximum-likelihood tree reconstructed from 16S rDNA sequences of known “*Ca*. Liberibacter” species and those detected in this study. The phylogenetic analysis was conducted with the K2 + G + I model (1,000 replicates of bootstrap analysis). The sequence of *Sinorhizobium alkalisoli* YIC4027^T^ was included as an outgroup. The “*Ca*. Liberibacter” sequences/strains detected in this study are indicated with bold fonts, and photos of their associated psyllid species are shown on the right. The psyllid species’ affiliated families are also indicated.

A total of five liberibacter-positive psyllid species were identified, namely *Calophya nigridorsalis* (Calophyidae), *Homotoma radiata* (Carsidaridae), *Cacopsylla tobirae* (Psyllidae), *Epipsylla albolineata* (Psyllidae), and *Trioza quadrimaculata* (Triozidae), with corresponding detection rates of 6.25%, 30%, 60%, 100%, and 25% (Table S2). After aligning the sequences with ClustalW (1,327 nt in length), sequences of the shared interval (1,299–1,320 nt; accession nos. PV292296–PV292300) were subjected to BLASTn searches against public databases (standard nr/nt, microbial complete genome, and microbial [“*Ca*. Liberibacter”] draft genome). Among the five detected “*Ca*. Liberibacter” strains, the strain detected in *C. tobirae* shared 99.92% sequence identity (1,302/1,303 nt) with a “*Ca*. Liberibacter europaeus” strain infecting the psyllid *Cacopsylla oluanpiensis* in Taiwan (16), whereas the sequence detected in *E. albolineata* was highly similar (99.69%; 1,295/1,299 nt) to that detected in a *Zanthoxylum* species (a Rutaceae plant) in Bhutan, which hosts two psyllids, a *Cacopsylla* species and *Cornopsylla rotundiconis* (20). For “*Ca*. Liberibacter” strains detected in *C. nigridorsalis*, *H. radiata*, and *T. quadrimaculata*, their sequence identities with the top BLASTn hits (across the searched databases) were 96.47%, 97.70%, and 98.49%, respectively (Tables S3 to S5), lower than generally accepted thresholds for species differentiation (21). A maximum-likelihood tree (K2 + G + I model; 1,000 bootstrap replicates) was reconstructed based on a ClustalW alignment including the detected strains, their top BLASTn hits, and strains of known “*Ca*. Liberibacter” species (Table S6) using MEGA12 (22). If a strain had tied top hits from the same species, only one representative hit was included. The results were consistent with those from the BLASTn searches, with long branches separating strains in *C. nigridorsalis*, *H. radiata*, *T. quadrimaculata*, and their top BLASTn hits or recognized “*Ca*. Liberibacter” species (Fig. 1).

“*Ca*. Liberibacter” strains have been detected in psyllids belonging to families including Aphalaridae (23), Carsidaridae (17, 24), Psyllidae (7, 16), and Triozidae (25). The findings from this work represent one of the first pieces of evidence showing that a member of the Calophyidae family harbors “*Ca*. Liberibacter”. Importantly, BLASTn and phylogenetic tree analyses both suggested that several strains detected in this study may belong to novel species, indicating that the diversity of “*Ca*. Liberibacter” in nature may be much greater than currently known.

It is crucial to note that in species like *C. nigridorsalis* and *T. quadrimaculata*, the detection rates were low. Studies have shown that “*Ca*. Liberibacter” infection rates can be population dependent (16), and some populations in this study could have had relatively low infection prevalence. Additionally, low-titer “*Ca*. Liberibacter” infections that are undetectable by PCR may also occur. It is possible that these factors and limited sample sizes have prohibited the identification of more “*Ca*. Liberibacter” among the psyllid species examined, and the prevalence of “*Ca*. Liberibacter” in Psylloidea could be greater than what has been shown. Nevertheless, this study is among the broadest “*Ca*. Liberibacter” surveys to date, in terms of both psyllid taxa and their plant hosts’ diversity. These results open opportunities for expanding knowledge on the evolutionary relationship between “*Ca*. Liberibacter” and psyllids.

## Data Availability

The sequences obtained in this study have been deposited in GenBank under the accession numbers PV292296, PV292297, PV292298, PV292299, and PV292300.

## SUPPLEMENTAL MATERIAL

Supplemental material spectrum.01228-25-s0001.pdf Supplemental methods; Tables S1 to S6.

ASM does not own the copyrights to Supplemental Material that may be linked to, or accessed through, an article. The authors have granted ASM a non-exclusive, world-wide license to publish the Supplemental Material files. Please contact the corresponding author directly for reuse.
