# Supplementary material for: Screening of diverse Psylloidea species in Taiwan reveals the presence of both known and potentially novel “Candidatus Liberibacter” species in multiple psyllid lineages
Source: Microbiol Spectr. 2025 Jun 30;13(8):e01228-25. doi: 10.1128/spectrum.01228-25 (PMC12323371; doi:10.1128/spectrum.01228-25)
Supplement: Supplemental material — Supplemental methods; Tables S1 to S6. [file spectrum.01228-25-s0001.pdf]

SUPPLEMENTAL MATERIAL

Supplemental methods

DNA extraction. Each psyllid was rinsed with 70% ethanol and then twice with sterile water. Subsequently, 180 µL of buffer ATL included in the DNeasy Blood and Tissue Kit (Qiagen, Inc., Valencia, CA) was added to the sample, and the insect was homogenized with a sterile micropestle. The processed sample was subjected to further extraction procedures following the manufacturer's instructions.

Polymerase chain reaction tests targeting Hexapoda mitochondrial 16S rDNA. Assays using primers Chiar16SF and Chiar16SR (1) (Table S1) targeting Hexapoda mitochondrial 16S rDNA were conducted to confirm the quality of the psyllid DNA. All polymerase chain reaction tests in this study were conducted using the GoTaq Green Master Mix (Promega, Madison, WI). Each 20 µL reaction included 0.5 µM of each primer and 1 µL of psyllid DNA (on average 83.5 ng). The PCR conditions were 95°C for 3 min, 35 cycles of 95°C for 30 S, 47°C for 30 S, 72°C for 30 S, and a final extension step at 72°C for 5 min. All results were confirmed by gel electrophoresis.

Polymerase chain reaction tests targeting '*Ca*. Liberibacter' 16S rDNA. Psyllid DNA samples were subjected to PCR using primers LG774F (2) and LG1463Rm (Table S1). LG1463Rm was modified from LG1463R, a reverse primer previously designed with LG774F (2). Modifications were made according to the alignment of 16S rDNA sequences of '*Ca*. Liberibacter asiaticus' (CP004005, CP001677), '*Ca*. Liberibacter africanus' (CP004021, CP054879), '*Ca*. Liberibacter americanus' (CP006604), '*Ca*. Liberibacter europaeus' (OQ701538, PSQJ01000002), '*Ca.* Liberibacter solanacearum' (PKRU0200000000), '*Ca.* Liberibacter psyllaurous' (EU812556), '*Ca.* Liberibacter brunswickensis' (JAAEJX010000019), '*Ca.* Liberibacter ctenarytainae' (SEOL01000017), *Liberibacter crescens* (NC_019907). The expected amplicon size for LG774F/LG1463Rm was approximately 690 bp. The PCR assays were conducted in 25 µL reactions, each including 0.4 µM of each primer with the same amounts of psyllid DNA as in tests using Chiar16SF/Chiar16SR. The PCR conditions were 95°C for 3 min, 35 cycles of 95°C for 30 S, 62°C for 30 S, 72°C for 1 min, and a final extension step at 72°C for 5 min.

After determining the PCR results using gel electrophoresis, DNA samples producing amplicons with the expected size were further amplified using primers 27F (3) and LG1463Rm to obtain near-full-length 16S rDNA sequences. These tests were also conducted in 25 µL reactions. Each reaction included 0.4 µM of each primer and the same amounts of template DNA as in previous tests. Since 27F and LG1463Rm have differing optimal annealing temperatures, gradient PCR assay using DNA samples from adult *Macrohomotoma gladiata* [a '*Ca*. Liberibacter'-positive psyllid species; (4)] were used to determine the efficacy of the assay and identify an ideal annealing temperature. The PCR conditions determined for these primers were 95°C for 3 min, 35 cycles of 95°C for 30 S, 60°C for 30 S, 72 °C for 1 min 30 S, and a final extension step at 72 °C for 5 min. The PCR results were examined using gel electrophoresis and the amplicons produced were recovered using a Zymoclean Gel DNA Recovery Kit (Zymo Research, Irvine, CA) and subjected to Sanger sequencing.

Screening of clone libraries. For samples that produced faint bands after amplification by 27F and LG1463Rm, the PCR products were cloned using pGEM-T Easy Vector System (Promega, Madison, WI) into *Escherichia coli* strain JM109 and subjected to blue-white screening. For each psyllid species that underwent the clone screening process, 6 to 29 white colonies were randomly selected for colony PCR by LG774F/M13-F and LG774F/M13-R (Table S1). The reaction volume for these tests was 20 µL. Each reaction contained 0.1 µM of each primer. After gel electrophoresis, products with the expected sizes were recovered and sequenced. The obtained sequences were searched using BLASTn to determine whether they were gene sequences of '*Ca*. Liberibacter'.

Supplemental tables

Table S1 Information of the primers used in this study

Target of primers	Name and sequence (5' to 3') of primer	Reference or source
16S rDNA of '*Ca*. Liberibacter'	LG774F: GTAAACGATGAGTGCTAGCTGTTGGG	(2)
	LG1463Rm[1]: CTGACC**Y**TACCGTGGCCGG	
Bacterial 16S rDNA	27F: AGAGTTTGATCMTGGCTCAG	(3)
Fragment in pGEM-T Easy Vector	M13-F: CACGACGTTGTAAAACGAC	(5)
(including cloned 16S rDNA sequences)	M13-R: GGATAACAATTTCACACAGG	

[1]LG1463Rm was modified from LG1463R (2); the modified nucleotide is indicated with bold font.

Table S2 Information on the detection rates and the number of samples successfully sequenced for the '*Candidatus* Liberibacter'-positive psyllid species in this study

Psyllid family	Psyllid species	No. of samples tested for the psyllid species	No. of '*Ca*. Liberibacter'-positive samples (detection rate)[1]	Gender of '*Ca*. Liberibacter'-positive psyllid(s)/sample(s)	No. of samples whose PCR products were successfully sequenced[2]
Calophyidae	*Calophya nigridorsalis*	16	1 (6.25%)	Male	1
Carsidaridae	*Homotoma radiata*	10	3 (30%)	Male and female	1
Psyllidae	*Cacopsylla tobirae*	5	3 (60%)	Female	2
	Epipsylla albolineata	11	11 (100%)	Male and female	5
Triozidae	*Trioza quadrimaculata*	8	2 (25%)	Female	1

[1]Number of samples of a psyllid species that successfully amplified the '*Ca*. Liberibacter'-specific amplicon with LG774F/LG1463Rm.
[2]Only DNA samples with intense bands detected in assays using LG774F/LG1463Rm were successfully sequenced. DNA samples that were depleted after previous assays were not sequenced.

Table S3 Top BLASTn hits of near-full-length 16S rDNA sequences from '*Candidatus* Liberibacter' strains detected in this study against NCBI's nucleotide collection (nr/nt) database[1,2]

Psyllid species in which '*Ca.* Liberibacter' was detected (Length of sequence used for BLASTn)	GenBank accession no.	Description (note)	Sequence identity (%)/ coverage (%)
Cacopsylla tobirae (1,303 nt)	OQ701538	Uncultured *Candidatus* Liberibacter sp. clone PLM 16S ribosomal RNA gene, partial sequence	99.92/100
	JX244259	*Candidatus* Liberibacter europaeus isolate BrS 16S ribosomal RNA gene, partial sequence; 16S-23S ribosomal RNA intergenic spacer, tRNA-Ile and tRNA-Ala genes, complete sequence; and 23S ribosomal RNA gene, partial sequence	99.69/100
	JX244258	*Candidatus* Liberibacter europaeus isolate Psy6 16S ribosomal RNA gene, partial sequence; 16S-23S ribosomal RNA intergenic spacer, tRNA-Ile and tRNA-Ala genes, complete sequence; and 23S ribosomal RNA gene, partial sequence	99.69/100
	FN678792	*Candidatus* Liberibacter sp. NR-01 partial 16S rRNA gene, strain NR-01	99.69/100
	CP006604	*Candidatus* Liberibacter americanus str. Sao Paulo, complete genome	96.09/100
	EU754742	*Candidatus* Liberibacter americanus strain Sao Paulo 16S ribosomal RNA gene, partial sequence; 16S-23S ribosomal RNA intergenic spacer, 23S ribosomal RNA and *glpK* genes, complete sequence; glycerol kinase gene, complete cds; and 5S ribosomal RNA gene, complete sequence	96.09/100
	EU921625	*Candidatus* Liberibacter americanus strain Sao Paulo-40 16S ribosomal RNA gene, partial sequence	96.09/100
	EU921623	*Candidatus* Liberibacter americanus strain Sao Paulo-275 16S ribosomal RNA gene, partial sequence	96.09/100
	AY742824	*Candidatus* Liberibacter americanus 16S ribosomal RNA gene, partial sequence	96.09/100
Calophya nigridorsalis (1,303 nt)	KX768754	*Candidatus* Liberibacter ctenarytainae 16S ribosomal RNA gene, partial sequence	96.32/100
	FN678792	*Candidatus* Liberibacter sp. NR-01 partial 16S rRNA gene, strain NR-01	95.71/100
	KX990287	*Candidatus* Liberibacter africanus subsp. zanthoxyli isolate Knysna 16S ribosomal RNA gene, partial sequence	95.46/100
	JX244259	*Candidatus* Liberibacter europaeus isolate BrS 16S ribosomal RNA gene, partial sequence; 16S-23S ribosomal RNA intergenic spacer, tRNA-Ile and tRNA-Ala genes, complete sequence; and 23S ribosomal RNA gene, partial sequence	95.63/100
	JX244258	*Candidatus* Liberibacter europaeus isolate Psy6 16S ribosomal RNA gene, partial sequence; 16S-23S ribosomal RNA intergenic spacer, tRNA-Ile and tRNA-Ala genes, complete sequence; and 23S ribosomal RNA gene, partial sequence	95.63/100
Epipsylla albolineata (1,299 nt)	KX768754	*Candidatus* Liberibacter ctenarytainae 16S ribosomal RNA gene, partial sequence	96.01/100
	MN203627	Uncultured *Candidatus* Liberibacter sp. clone #51 16S ribosomal RNA gene, partial sequence	99.65/89
	NR_102476	*Liberibacter crescens* strain BT-1 16S ribosomal RNA, complete sequence	95.85/100
	CP010522	*Liberibacter crescens* strain BT-0, complete genome	95.85/100
	CP003789	*Liberibacter crescens* BT-1, complete genome	95.85/100
Homotoma radiata (1,301 nt)	CP006604	*Candidatus* Liberibacter americanus str. Sao Paulo, complete genome	97.70/100
	EU754742	*Candidatus* Liberibacter americanus strain Sao Paulo 16S ribosomal RNA gene, partial sequence; 16S-23S ribosomal RNA intergenic spacer, 23S ribosomal RNA and *glpK* genes, complete sequence; glycerol kinase gene, complete cds; and 5S ribosomal RNA gene, complete sequence	97.70/100
	EU921625	*Candidatus* Liberibacter americanus strain Sao Paulo-40 16S ribosomal RNA gene, partial sequence	97.70/100

Table S3 (continued)

	EU921623	*Candidatus* Liberibacter americanus strain Sao Paulo-275 16S ribosomal RNA gene, partial sequence	97.70/100
	AY742824	*Candidatus* Liberibacter americanus 16S ribosomal RNA gene, partial sequence	97.70/100
	FJ914621	*Candidatus* Liberibacter sp. Sao_Paulo-2 16S ribosomal RNA gene, partial sequence; tRNA-Ile, tRNA-Ala, and 23S ribosomal RNA genes, complete sequence; glycerol kinase gene, complete cds; and 5S ribosomal RNA and tRNA-Met genes, complete sequence	97.69/100
	OQ725634	*Candidatus* Liberibacter americanus strain SJ1 16S ribosomal RNA gene, partial sequence	97.31/100
	EU921624	*Candidatus* Liberibacter americanus strain Sao Paulo-PW-49 16S ribosomal RNA gene, partial sequence	97.31/100
Trioza quadrimaculata (1,320 nt)	PP808504	*Candidatus* Liberibacter psyllaurous clone BC 16S ribosomal RNA gene, partial sequence	98.49/100
	EU921626	*Candidatus* Liberibacter sp. Garden-city-KS-1 16S ribosomal RNA gene, partial sequence	98.49/100
	KX431890	*Candidatus* Liberibacter solanacearum isolate 16sB 16S ribosomal RNA, tRNA-Ile, tRNA-Ala, 23S ribosomal RNA, 5S ribosomal RNA, and tRNA-Met genes, complete sequence	98.41/100
	KX431889	*Candidatus* Liberibacter solanacearum isolate 16sA 16S ribosomal RNA, tRNA-Ile, tRNA-Ala, 23S ribosomal RNA, 5S ribosomal RNA, and tRNA-Met genes, complete sequence	98.41/100
	EU935004	*Candidatus* Liberibacter solanacearum isolate NZ083338 16S ribosomal RNA gene and 16S-23S ribosomal RNA intergenic spacer, partial sequence; and tRNA-Ile and tRNA-Ala genes, complete sequence	98.41/100
	EU812559	*Candidatus* Liberibacter psyllaurous isolate PRR1 16S ribosomal RNA gene, partial sequence; 16S-23S ribosomal RNA intergenic spacer, complete sequence; and 23S ribosomal RNA gene, partial sequence	98.41/100
	EU812558	*Candidatus* Liberibacter psyllaurous isolate Tom100 16S ribosomal RNA gene, partial sequence; 16S-23S ribosomal RNA intergenic spacer, complete sequence; and 23S ribosomal RNA gene, partial sequence	98.41/100
	EU812556	*Candidatus* Liberibacter psyllaurous isolate Tx15 16S ribosomal RNA gene, partial sequence; 16S-23S ribosomal RNA intergenic spacer, complete sequence; and 23S ribosomal RNA gene, partial sequence	98.41/100
	EU834130	*Candidatus* Liberibacter solanacearum isolate NZ082226 16S ribosomal RNA gene and 16S-23S ribosomal RNA intergenic spacer, partial sequence; and tRNA-Ile and tRNA-Ala genes, complete sequence	98.41/100

[1]Standard nucleotide BLAST results.

[2]Hits with the top 3 maximum scores are listed.

Table S4 Top BLASTn hits of near-full-length 16S rDNA sequences from '*Candidatus* Liberibacter' strains detected in this study against NCBI's microbial complete genomes database[1,2]

Psyllid species in which '*Ca*. Liberibacter' was detected (Length of sequence used for BLASTn)	GenBank accession no.	Description (note)	Sequence identity (%)/ coverage (%)
Cacopsylla tobirae (1,303 nt)	NC_022793	*Candidatus* Liberibacter americanus str. Sao Paulo, complete sequence	96.09/100
	NZ_CP146613	*Candidatus* Liberibacter africanus strain Zim chromosome, complete genome	95.15/98
	NZ_CP004021	*Candidatus* Liberibacter africanus PTSAPSY chromosome, complete genome	95.15/98
	NC_014774	*Candidatus* Liberibacter solanacearum CLso-ZC1, complete sequence	94.61/99
Calophya nigridorsalis (1,303 nt)	NC_022793	*Candidatus* Liberibacter americanus str. Sao Paulo, complete sequence	95.63/100
	NZ_CP146613	*Candidatus* Liberibacter africanus strain Zim chromosome, complete genome	95.38/100
	NZ_CP004021	*Candidatus* Liberibacter africanus PTSAPSY chromosome, complete genome	95.38/100
	NC_014774	*Candidatus* Liberibacter solanacearum CLso-ZC1, complete sequence	94.70/100
Epipsylla albolineata (1,299 nt)	NC_019907	*Liberibacter crescens* BT-1, complete sequence	95.85/100
	NZ_CP010522	*Liberibacter crescens* strain BT-0 chromosome, complete genome	95.85/100
	NZ_CP146613	*Candidatus* Liberibacter africanus strain Zim chromosome, complete genome	95.53/100
	NZ_CP004021	*Candidatus* Liberibacter africanus PTSAPSY chromosome, complete genome	95.53/100
	NC_022793	*Candidatus* Liberibacter americanus str. Sao Paulo, complete sequence	96.02/100
Homotoma radiata (1,301 nt)	NC_022793	*Candidatus* Liberibacter americanus str. Sao Paulo, complete sequence	97.70/100
	NC_019907	*Liberibacter crescens* BT-1, complete sequence	95.78/100
	NZ_CP010522	*Liberibacter crescens* strain BT-0 chromosome, complete genome	95.78/100
	NZ_CP146613	*Candidatus* Liberibacter africanus strain Zim chromosome, complete genome	95.30/100
	NZ_CP004021	*Candidatus* Liberibacter africanus PTSAPSY chromosome, complete genome	95.30/100
Trioza quadrimaculata (1,320 nt)	NC_014774	*Candidatus* Liberibacter solanacearum CLso-ZC1, complete sequence	98.34/100
	NZ_CP146613	*Candidatus* Liberibacter africanus strain Zim chromosome, complete genome	97.73/100
	NZ_CP004021	*Candidatus* Liberibacter africanus PTSAPSY chromosome, complete genome	97.73/100
	NZ_CP145497	*Candidatus* Liberibacter asiaticus strain 9PA chromosome, complete genome	97.35/100
	NZ_CP054558	*Candidatus* Liberibacter asiaticus isolate CoFLP chromosome, complete genome	97.35/100
	NZ_AP014595	*Candidatus* Liberibacter asiaticus str. Ishi-1 chromosome, complete genome	97.35/100
	NZ_CP010804	*Candidatus* Liberibacter asiaticus strain A4 chromosome, complete genome	97.35/100
	NZ_CP040636	*Candidatus* Liberibacter asiaticus isolate JRPAMB1 chromosome, complete genome	97.35/100
	NZ_CP159585	*Candidatus* Liberibacter asiaticus strain GDCH12 chromosome, complete genome	97.35/100
	NC_020549	*Candidatus* Liberibacter asiaticus str. gxpsy, complete sequence	97.35/100
	NZ_CP041385	*Candidatus* Liberibacter asiaticus isolate TaiYZ2 chromosome, complete genome	97.35/100
	NZ_CP118771	*Candidatus* Liberibacter asiaticus strain YNBC chromosome, complete genome	97.35/100

Table S4 (continued)

	NZ_CP019958	*Candidatus* Liberibacter asiaticus strain JXGC chromosome, complete genome	97.35/100
	NZ_CP118922	*Candidatus* Liberibacter asiaticus strain GDCZ chromosome, complete genome	97.35/100
	NC_012985	*Candidatus* Liberibacter asiaticus str. psy62, complete sequence	97.35/100
	NZ_CP100417	*Candidatus* Liberibacter asiaticus isolate PYN chromosome, complete genome	97.35/100
	NZ_CP100754	*Candidatus* Liberibacter asiaticus isolate PGD chromosome, complete genome	97.35/100

[1]Microbial nucleotide BLAST results.

[2]Hits with the top 3 maximum scores are listed.

Table S5 Top BLASTn hits of near-full-length 16S rDNA sequences from '*Candidatus* Liberibacter' strains detected in this study against NCBI's microbial draft genomes database[1,2]

Psyllid species in which '*Ca*. Liberibacter' was detected (Length of sequence used for BLASTn)	GenBank accession no.	Description (note)	Sequence identity (%)/ coverage (%)
Cacopsylla tobirae (1,303 nt)	QEYS01000023	MAG: *Candidatus* Liberibacter europaeus isolate ASUK1 CLeu_ASUK1_23, whole genome shotgun sequence	99.69/100
	QEYS01000011	MAG: *Candidatus* Liberibacter europaeus isolate ASUK1 CLeu_ASUK1_11, whole genome shotgun sequence	99.69/100
	QEYS01000003	MAG: *Candidatus* Liberibacter europaeus isolate ASUK1 CLeu_ASUK1_03, whole genome shotgun sequence	99.69/100
	PSQJ01000010	MAG: *Candidatus* Liberibacter europaeus isolate ASNZ1 CLeu_NZ1_10, whole genome shotgun sequence	99.69/100
	PSQJ01000002	MAG: *Candidatus* Liberibacter europaeus isolate ASNZ1 CLeu_NZ1_02, whole genome shotgun sequence	99.69/100
	PSQJ01000001	MAG: *Candidatus* Liberibacter europaeus isolate ASNZ1 CLeu_NZ1_01, whole genome shotgun sequence	99.69/100
	AOFG01000011	*Candidatus* Liberibacter americanus PW_SP full_22_00011, whole genome shotgun sequence	95.93/100
	AOFG01000004	*Candidatus* Liberibacter americanus PW_SP full_22_00004, whole genome shotgun sequence	95.93/100
	AOFG01000002	*Candidatus* Liberibacter americanus PW_SP full_22_00002, whole genome shotgun sequence	95.86/100
Calophya nigridorsalis (1,303 nt)	JACETX010000057	Liberibacter sp. Z1 NODE_6742_length_5319_cov_84.499618, whole genome shotgun sequence	96.47/100
	SEOL01000017	MAG: *Candidatus* Liberibacter ctenarytainae isolate Oxford CLct_Ox_contig17, whole genome shotgun sequence	96.32/100
	JAAEJX010000019	*Candidatus* Liberibacter brunswickensis strain Asol15 NODE_20_length_5819_cov_760.929346, whole genome shotgun sequence	95.54/100
Epipsylla albolineata (1,299 nt)	JACETX010000057	Liberibacter sp. Z1 NODE_6742_length_5319_cov_84.499618, whole genome shotgun sequence	99.69/100
	SEOL01000017	MAG: *Candidatus* Liberibacter ctenarytainae isolate Oxford CLct_Ox_contig17, whole genome shotgun sequence	96.01/100
	QEYS01000023	MAG: *Candidatus* Liberibacter europaeus isolate ASUK1 CLeu_ASUK1_23, whole genome shotgun sequence	96.17/98
	QEYS01000011	MAG: *Candidatus* Liberibacter europaeus isolate ASUK1 CLeu_ASUK1_11, whole genome shotgun sequence	96.17/98
	QEYS01000003	MAG: *Candidatus* Liberibacter europaeus isolate ASUK1 CLeu_ASUK1_03, whole genome shotgun sequence	96.17/98
	PSQJ01000010	MAG: *Candidatus* Liberibacter europaeus isolate ASNZ1 CLeu_NZ1_10, whole genome shotgun sequence	96.17/98
	PSQJ01000002	MAG: *Candidatus* Liberibacter europaeus isolate ASNZ1 CLeu_NZ1_02, whole genome shotgun sequence	96.17/98
	PSQJ01000001	MAG: *Candidatus* Liberibacter europaeus isolate ASNZ1 CLeu_NZ1_01, whole genome shotgun sequence	96.17/98
Homotoma radiata (1,301 nt)	AOFG01000011	*Candidatus* Liberibacter americanus PW_SP full_22_00011, whole genome shotgun sequence	97.54/100
	AOFG01000004	*Candidatus* Liberibacter americanus PW_SP full_22_00004, whole genome shotgun sequence	97.54/100
	AOFG01000002	*Candidatus* Liberibacter americanus PW_SP full_22_00002, whole genome shotgun sequence	97.47/100
	JACETX010000057	Liberibacter sp. Z1 NODE_6742_length_5319_cov_84.499618, whole genome shotgun sequence	96.85/100
Trioza quadrimaculata (1,320 nt)	JNVH01000030	*Candidatus* Liberibacter solanacearum strain R1 contig30, whole genome shotgun sequence	98.49/100
	JBFPDM010000016	MAG: *Candidatus* Liberibacter psyllaurous isolate BC-3 k141_14619, whole genome shotgun sequence	98.49/100

Table S5 (continued)

	CAXYJN010000017	*Candidatus* Liberibacter solanacearum isolate FR9j genome assembly, contig: LSO9J_Contig_25, whole genome shotgun sequence	98.49/100
	CAXYJM010000016	*Candidatus* Liberibacter solanacearum isolate FR4a genome assembly, contig: LSO4A_Contig_24, whole genome shotgun sequence	98.49/100
	CAXYJL010000018	*Candidatus* Liberibacter solanacearum isolate FR10f genome assembly, contig: LSO10F_Contig_26, whole genome shotgun sequence	98.49/100
	CAXYJK010000019	*Candidatus* Liberibacter solanacearum isolate FR2f genome assembly, contig: LSO2F_Contig_27, whole genome shotgun sequence	98.49/100
	CAXYJJ010000015	*Candidatus* Liberibacter solanacearum isolate FR12e genome assembly, contig: LSO12E_Contig_23, whole genome shotgun sequence	98.49/100
	LWEB01000002	*Candidatus* Liberibacter solanacearum strain FIN114 contig_2, whole genome shotgun sequence	98.41/100
	LVWB01000013	*Candidatus* Liberibacter solanacearum strain FIN111 111_contig13, whole genome shotgun sequence	98.41/100
	LVWB01000008	*Candidatus* Liberibacter solanacearum strain FIN111 111_contig8, whole genome shotgun sequence	98.41/100
	LLVZ01000011	*Candidatus* Liberibacter solanacearum strain RSTM RSTM.Contig11, whole genome shotgun sequence	98.41/100
	JQIG01000007	*Candidatus* Liberibacter solanacearum strain HenneA LsoUSA1_contig7, whole genome shotgun sequence	98.41/100
	JQIG01000005	*Candidatus* Liberibacter solanacearum strain HenneA LsoUSA1_contig5, whole genome shotgun sequence	98.41/100
	JQIG01000001	*Candidatus* Liberibacter solanacearum strain HenneA LsoUSA1_contig1, whole genome shotgun sequence	98.41/100
	JMTK01000005	*Candidatus* Liberibacter solanacearum strain LsoNZ1 CLso_NZ1_contig5, whole genome shotgun sequence	98.41/100
	JMTK01000002	*Candidatus* Liberibacter solanacearum strain LsoNZ1 CLso_NZ1_contig2, whole genome shotgun sequence	98.41/100
	JMTK01000001	*Candidatus* Liberibacter solanacearum strain LsoNZ1 CLso_NZ1_contig1, whole genome shotgun sequence	98.41/100
	JACEEQ010000009	MAG: *Candidatus* Liberibacter solanacearum isolate FINH40 FINH40_contig9, whole genome shotgun sequence	98.41/100
	JACEEQ010000004	MAG: *Candidatus* Liberibacter solanacearum isolate FINH40 FINH40_contig4, whole genome shotgun sequence	98.41/100
	JNVH01000097	*Candidatus* Liberibacter solanacearum strain R1 contig97, whole genome shotgun sequence	98.34/100

[1]Microbial nucleotide BLAST results.

[2]Hits with the top 3 maximum scores are listed.

Table S6 Information of the '*Candidatus* Liberibacter' sequences that were retrieved from GenBank and included in the phylogenetic analysis

**Species**	**Strain/isolate/clone**	**Host/source of bacterium**	**Locality**	**Accession number**
'*Candidatus* Liberibacter asiaticus'	psy62	*Diaphorina citri*	U.S.A.	CP001677
	gxpsy	*Diaphorina citri*	China	CP004005
	Ishi-1	*Diaphorina citri*	Japan	AP014595
'*Ca.* Liberibacter americanus'	Sao Paulo	*Citrus sinensis*	Brazil	CP006604
'*Ca.* Liberibacter africanus'	PTSAPSY	*Trioza erytreae*	South Africa	CP004021
	Ang37	*Citrus reticulata*	Angola	CP054879
	Zim	*Citrus* × *limon*	Zimbabwe	CP146613
'*Ca.* Liberibacter solanacearum'	ISR100	*Bactericera trigonica*	Israel	PKRU02000006
('*Ca.* Liberibacter psyllaurous')	Tx15	*Bactericera cockerelli*	U.S.A.	EU812556
	BC	*Bactericera cockerelli*	n.a.[1]	PP808504
	CLso-ZC1	*Bactericera cockerelli*	U.S.A.	NC_014774
'*Ca.* Liberibacter europaeus'	PLM	*Cacopsylla oluanpiensis*	Taiwan	OQ701538
	NR-01	*Cacopsylla pyri*	Italy	FN678792
	ASNZ1	*Arytainilla spartiophila*	New Zealand	PSQJ01000002
'*Ca.* Liberibacter brunswickensis'	Asol15	*Acizzia solanicola*	Australia	JAAEJX010000019
'*Ca.* Liberibacter ctenarytainae'	Oxford	*Ctenarytaina fuchsiae*	New Zealand	SEOL01000017
Unnamed '*Ca.* Liberibacter' sp.	TC9	*Macrohomotoma gladiata*	Taiwan	OP159054
Unnamed '*Ca.* Liberibacter' sp.	Z1	*Zanthoxylum* sp.	Bhutan	JACETX010000057
Liberibacter crescens	BT-1^T	*Carica stipulata* × *C. pubescens*	Puerto Rico	NC_019907
Sinorhizobium alkalisoli (outgroup)	YIC4027^T	*Sesbania cannabina*	China	KX668272

[1]n.a.: not available

References

1. Marquina D, Andersson AF, Ronquist F. 2018. New mitochondrial primers for metabarcoding of insects, designed and evaluated using *in silico* methods. Mol Ecol Resour 19:90-104.
2. Morris J, Shiller J, Mann R, Smith G, Yen A, Rodoni B. 2017. Novel '*Candidatus* Liberibacter' species identified in the Australian eggplant psyllid, *Acizzia solanicola*. Microb Biotechnol 10:833-844.
3. Lane DJ. 1991. 16S/23S rRNA sequencing, p 115-148. *In* Stackebrandt E, Goodfellow M (ed), Nucelic acid techniques in bacterial systematics. Wiley & Sons Ltd., Chichester, UK.
4. Lin FY, Lee S, Liao YC, Yang MM, Chu CC. 2022. Infection patterns of a Liberibacter associated with *Macrohomotoma gladiata*, a psyllid feeding on *Ficus microcarpa*. Microbiol Spectr 10:e0361422.
5. Lada H, Taylor AC. 2009. Polymorphic nuclear markers for aquatic macroinvertebrates *Anisops hackeri*, *Micronecta gracilis* and *Necterosoma wollastoni*. Conserv Genet 10:1625-1627.
